# Sanatoria for the People

**Published:** 1911-10-28

**Authors:** 


### SANATORIA: THEIR USE AND ABUSE.

(Contributions, experiences and questions relating to Sanatoria, their Administration and Work are invited.)

### Sanatoria for the People.

WHAT THEY CAN AND CANNOT ACCOMPLISH.

For the last ten years the particular form of outdoor treatment of tuberculosis which is associated with the word sanatorium has fascinated public interest to a degree which is both natural and praiseworthy. There are few families in which some member or some relative has not been affected by this omnipresent scourge, and consequently there are few intelligent people who do not take an interest in a method of repairing its ravages about which such glowing accounts have been published within the last decade. Lately there has been in some medical circles a swing of the pendulum, and sanatoria have been stated to have failed to fulfil expectations formed of them. Unquestionably sanatoria do not cure every case of consumption, nor are the cures that they do produce always permanent. But there are other causes for that besides any inherent default of sanatorium treatment. The sanatorium is a link in a chain of measures necessary before consumption can be eradicated or even controlled; it is a strong link and a necessary link, certainly, but it is not the whole chain.

Hitherto sanatoria have been organised by private enterprise: either as commercial enterprises catering for patients sufficiently well off to pay adequate fees; or as institutions endowed by the charitable, and accepting from their patients either a part only of the cost of maintenance or nothing at all. Now sanatoria cannot ever be cheap, although they can be managed at a less cost per bed for buildings and upkeep than the majority of town hospitals. And hitherto the supply of sanatorium accommodation for the labouring classes has not equalled the demand, though a great deal has been done in this respect through the munificence of the charitably minded among the moneyed classes. Under the National Insurance Bill, however, as at present framed, it is intended to devote one and a half million sterling to the erection of sanatoria for the benefit of the insured; and further to devote a portion of the income derived under the Bill to the maintenance of these sanatoria when they are built. These provisions have stimulated anew the curiosity of the public in the matter of sanatoria, and have aroused afresh controversial discussions among the medical profession as to the real value of these institutions in the fight against consumption.

What is a sanatorium? What can it do? How much does it cost? These are questions which the well-informed layman has a right to ask his medical man. The answers, together with a great deal more information of an intensely interesting and practical nature, have been supplied in a remarkable book* just published by Mr. Garland and Dr. Lister, respectively Chairman of and Consulting Physician to the well-known National Sanatorium, Benenden, Kent. Laymen who are unacquainted with the technical details of the scientific aspects of tuberculosis, and are confused by the special pleadings of experts employing each some particular method in waging the fight, not unnaturally ask for some authoritative and comprehensible explanation of how it is proposed to conduct the war now that the State has engaged in the struggle, and what part in it is to be played by the Sanatorium. Members and officials of Friendly Societies are also asking questions and find it difficult to get an answer in a form which does not involve much research and lengthy reading. The object of this book is to elucidate, briefly and in popular language, what a working-class sanatorium is, and how it should be administered. The authors, it is hardly necessary to point out, have between them every qualification for this task; and they have carried it out in a manner which can elicit nothing but admiration and commendation.

Every side of the complicated problem is considered carefully in turn, and nothing is neglected. Thus one of the great defects of charitable sanatoria has turned out by experience to be this: that the patients become so accustomed to and enamoured of a life of loafing, that after their discharge they cannot be got to do any work. This means inevitably in the end privation due to insufficient food, and relapse follows. The solution of this problem is now being sought along the lines of graduated work for sanatorium patients, and it is hoped to turn them out (when cured) hard and fit to adopt manual labour. This labour, moreover, must be of a kind to conduce to health. It is useless to send the clerk back to his dusty office, or the stonemason back to his gritty yard. Herein lies much of the difficulty; for a large proportion of these patients are too stupid to realise that in farmwork or some similar occupation lies health and strength, but prefer to return to the noisome slums wherein their disease was conceived and propagated. For our own part, we confess that the crass obstinacy and stupidity of this type of patient seem often insurmountable. But the authors of this book take a sanguine view, and expound their system, which is based partly upon the experience of the Germans.

The construction of sanatoria, and the relations of sanatoria to other methods receive due attention: especial stress indeed is laid upon the organisation which needs to be built up to deal with consumption outside the scope of sanatoria. The industrial aspects of consumption are also exhaustively analysed, and tables of the latest available statistics are given. Finally, the bearings of the Insurance Bill upon the sanatorium question are fully examined. The authors are expert in their subject, and they have produced a popular account of it which should command a wide audience; we know of no better shillingsworth in this kind of literature, and do not doubt that the volume will prove of immense value to everyone who is interested in tuberculosis and in the National Insurance Bill.

* *Sanatoria for the People.* (The Scientific Press, Ltd. 1911. Price 1s. net.)

